# The automaticity of face perception is influenced by familiarity

**DOI:** 10.3758/s13414-017-1362-1

**Published:** 2017-07-05

**Authors:** Xiaoqian Yan, Andrew W. Young, Timothy J. Andrews

**Affiliations:** Department of Psychology, University of York, York, YO10 5DD UK

**Keywords:** Face, Race, Identity, Expression

## Abstract

In this study, we explore the automaticity of encoding for different facial characteristics and ask whether it is influenced by face familiarity. We used a matching task in which participants had to report whether the gender, identity, race, or expression of two briefly presented faces was the same or different. The task was made challenging by allowing nonrelevant dimensions to vary across trials. To test for automaticity, we compared performance on trials in which the task instruction was given at the beginning of the trial, with trials in which the task instruction was given at the end of the trial. As a strong criterion for automatic processing, we reasoned that if perception of a given characteristic (gender, race, identity, or emotion) is fully automatic, the timing of the instruction should not influence performance. We compared automaticity for the perception of familiar and unfamiliar faces. Performance with unfamiliar faces was higher for all tasks when the instruction was given at the beginning of the trial. However, we found a significant interaction between instruction and task with familiar faces. Accuracy of gender and identity judgments to familiar faces was the same regardless of whether the instruction was given before or after the trial, suggesting automatic processing of these properties. In contrast, there was an effect of instruction for judgments of expression and race to familiar faces. These results show that familiarity enhances the automatic processing of some types of facial information more than others.

The human face conveys a variety of different signals that are important for successful social interactions. The image of a face provides information about a person’s identity, gender, race, emotional state, and a range of other important attributes (Bruce & Young, 1986, 2012). The ease with which these properties appear to be detected and discriminated has led many researchers to suggest that face processing is automatic (Fiske & Neuberg, 1990; Freeman & Ambady, 2011; Öhman, 2002; Vuilleumier & Righart, 2011; for review, see Palermo & Rhodes, 2007). However, other theories and findings suggest that extraction of social information from faces can also involve some degree of top-down control (Macrae & Bodenhausen, 2000; Santos & Young, 2005).

As is often the case in psychology, this debate may in part reflect different criteria for automaticity. Four main interrelated attributes have been suggested to characterize automatic from controlled processes (Gawronski & Creighton, 2013; Logan, 1988; Moors, 2016; Schneider & Shiffrin, 1977; Shiffrin & Schneider, 1977). First, automatic processes will be fast. Second, they may be to some degree nonconscious. Third, automatic processes are involuntary. Fourth, automatic processes require limited attentional resources.

In terms of these criteria, human faces are detected and categorized more quickly than many other nonface objects and even animal faces. Electrophysiological studies have found that faces can be categorized within 100 ms of stimulus onset (Bentin et al., 1996; Liu, Harris, & Kanwisher, 2002; Oram & Perrett, 1992; Sugase, Yamane, Ueno, & Yawano, 1999), which is about 100 ms earlier than for other objects (Pegna, Khateb, Michel, & Landis, 2004). Behavioral studies have also found that the detection of faces in natural scenes occurs earlier for faces than for animals (Rousselet, Macé, & Fabre-Thorpe, 2003) and that the threshold for detecting upright faces is lower compared to inverted or scrambled faces (Besson et al., 2017; Purcell & Stewart, 1988). The detection and discrimination of emotional faces has also been shown to occur within 100 ms of stimulus onset (Eimer & Holmes, 2002; Pizzagalli, Regard, & Lehmann, 1999; Pourtois, Grandjean, Sander, & Vuilleumier, 2004). The ability to discriminate the identity of different individual faces takes longer, but some studies have sought to demonstrate that this can be achieved within 170 ms (Heisz, Watter, & Shedden, 2006; Jacques & Rossion, 2006; Liu et al., 2002; Sugase et al., 1999; although see Schweinberger, Pickering, Burton, & Kaufmann, 2002; Ewbank, Smith, Hancock, & Andrews, 2008).

A range of evidence also suggests that some information about faces can be processed in the absence of awareness. Behavioral studies in healthy participants have shown that faces that are not consciously perceived can nonetheless influence subsequent behavior. For example, the emotional expression from a briefly presented face that is not perceived can influence the subsequent perception of a neutral stimulus (Murphy & Zajonc, 1993; Rotteveel, de Groot, Geutskens, & Phaf, 2001). Neuroimaging studies have also shown that responses to different facial expressions can be discriminated in the brain even when the participants are not conscious of seeing the faces (Pasley, Mayes, & Schultz, 2004; Vuilleumier et al., 2002; Whalen et al., 1998; Williams, Morris, McGlone, Abbott, & Mattingley, 2004; although see Phillips et al., 2004; Pessoa, Japee, Sturman, & Ungerleider, 2006). Consistent with these neuroimaging studies, studies of the blindsight patient G.Y. have found above-chance discrimination of facial expression when faces are presented in the blind visual field (de Gelder, Vroomen, Pourtois, & Weiskrantz, 1999; Morris, de Gelder, Weiskrantz, & Dolan, 2001). Dissociations between awareness of recognition and behavioral or psychophysiological responses are also found in prosopagnosia, where differential responses to familiar compared to unfamiliar faces can be demonstrated despite the absence of conscious recognition (Bauer, 1984; Tranel & Damasio, 1985; de Haan, Young, & Newcombe, 1987; Young & Burton, 1999).

The extent to which faces are processed automatically can also be determined by the extent to which attention or task influences processing of the image. For example, evidence for automatic processing of the identities of familiar faces has been shown in face–name interference tasks, in which the ability to categorize a name is affected by whether a distractor face is congruent or incongruent with the correct response (Jenkins, Lavie, & Driver, 2003; Lavie, Ro, & Russell, 2003; Young et al. 1986a, b). Other studies have shown that performance on a task involving one facial dimension (e.g., identity) can be influenced by changes in an irrelevant dimension (e.g., expression), suggesting automatic processing of the unattended dimension (Martin et al., 2015; Schweinberger, Burton, & Kelly, 1999; Schweinberger & Soukup, 1998). Neuroimaging studies also provide support for the mandatory processing of faces. For example, the response in the amygdala is not different to attended or unattended fearful faces (Vuilleumier, Armony, Driver, & Dolan, 2001). However, although these findings suggest that some facial properties are processed irrespective of the task, other studies have shown that neural responses to faces can be modulated by attention (Downing, Liu, & Kanwisher, 2001; Eimer, 2000; Holmes, Vuilleumier, & Eimer, 2003; O’Craven, Downing, & Kanwisher, 1999). The importance of attention is also demonstrated by the enhanced discrimination of faces that are attended (Palermo & Rhodes, 2002; Reinitz, Morrissey, & Demb, 1994).

From this brief overview it is clear that the extent and limits of automaticity of face perception remain in some respects uncertain. A limitation of most previous studies of automaticity in face perception is that they have usually investigated only one dimension of the face. However, a range of evidence has suggested that different properties of the face may be processed independently (Duchaine & Yovel, 2015; Haxby, Hoffman, & Gobbini, 2000; Young & Bruce, 2011). So it remains unclear whether the level of automaticity varies across different facial dimensions. Another limitation in our understanding is that much of the key evidence is based on measures of brain imaging. Although these findings have helped our understanding, these measures are correlational in nature, and it is not always clear how patterns of brain response influence behavior. Finally, the majority of studies of automaticity have used unfamiliar faces (although see Gobbini et al., 2013; Jackson & Raymond, 2006; Tong & Nakayama, 1999; Visconti di Oleggio Castello & Gobbini, 2015), yet it is well-established that familiar faces are processed more effectively than unfamiliar faces (Burton, Jenkins, Hancock, & White, 2005; Hancock, Bruce & Burton, 2000; Jenkins & Burton, 2011). The question of whether the strong processing advantages that accrue to familiar faces extends beyond the perception of identity may therefore offer important insights into the nature and extent of automatic processing of different facial characteristics.

The aim of the current study, then, is to compare the automaticity of face perception across different characteristics and across unfamiliar and familiar faces. To achieve this, we introduce a new method that involves using a matching task in which participants report whether sequentially presented images of faces vary in gender, identity, race, or facial expression. The task is made difficult because the faces are briefly presented and because nonrelevant dimensions can vary across trials (for example, faces to be matched for same or different gender may also vary in identity, race, or expression). To contrast automatic with controlled processing, we compare performance when the dimension on which the faces have to be matched is given at the beginning of each trial, allowing the possibility of more controlled processing compared to when it is only given at the end of the trial, making performance therefore largely dependent on automatic processing of the preceding stimuli.

One of the main lessons of the extensive literature on automaticity is that it is not simply an “all or none” phenomenon. To interpret the extent of automatic processing of different facial characteristics, we therefore use two different criteria for automaticity. Our first criterion is that a fully automatic process will not be affected by whether the task is given at the beginning or at the end of the trial; that is, if a characteristic is processed automatically, you will not need to know in advance what to look for. Note that good performance when the task is only specified at the end of the trial will require that the participant can encode the target characteristic in both of the briefly presented faces, remember what was seen in the first face across the short interstimulus interval, and not be distracted from this memory by the second face in the sequence. It therefore represents a strong criterion that combines key properties of automaticity. Our second (weaker) criterion for automaticity is that performance when the task is given at the end of the trial will be above chance; this criterion accepts that some information that was automatically extracted from the first face may be lost across the brief interval in the matching trial but stipulates that this loss should not reduce performance to chance level. Using these criteria, we investigated automatic processing of the gender, race, identity and facial expression of unfamiliar faces in Experiment 1, and then compared automatic processing of these characteristics between familiar and unfamiliar faces in Experiment 2.

## Experiment 1

Experiment 1 investigated the automaticity of unfamiliar face perception using a matching task, in which the faces varied in gender (female or male), identity (same or different), race (Chinese or Caucasian) or emotional expression (happiness or surprise). The task instruction for what property to match (gender, identity, race, or emotion) in each trial was given to participants either at the beginning or the end of each trial.

### Participants

Twenty-eight Caucasian participants from the University of York were recruited for this experiment (20 females; mean age, 20.1 years). All participants gave their written consent prior to the experiment. The University of York Department of Psychology Ethics Committee approved the study.

### Images

Face stimuli were selected from two stimuli sets; the CAS-PEAL R1 face database (Gao et al., 2008), posed by Chinese models, and the Karolinska Directed Emotional Faces (KDEF) (Lundqvist, Flykt, & Öhman, 1998), posed by Caucasian models. In each set, images of 40 different identities with each identity posing two facial expressions (one happiness and one surprise) were selected, for each gender (female and male) and each race (Caucasian and Chinese), resulting in a total of 320 images. These 320 images were then rated and screened by three raters for each of the principal categories of interest (race, gender, and emotion) to choose 20 images each with the highest consistency among raters, resulting in a total of 160 images.

**Fig. 1:**
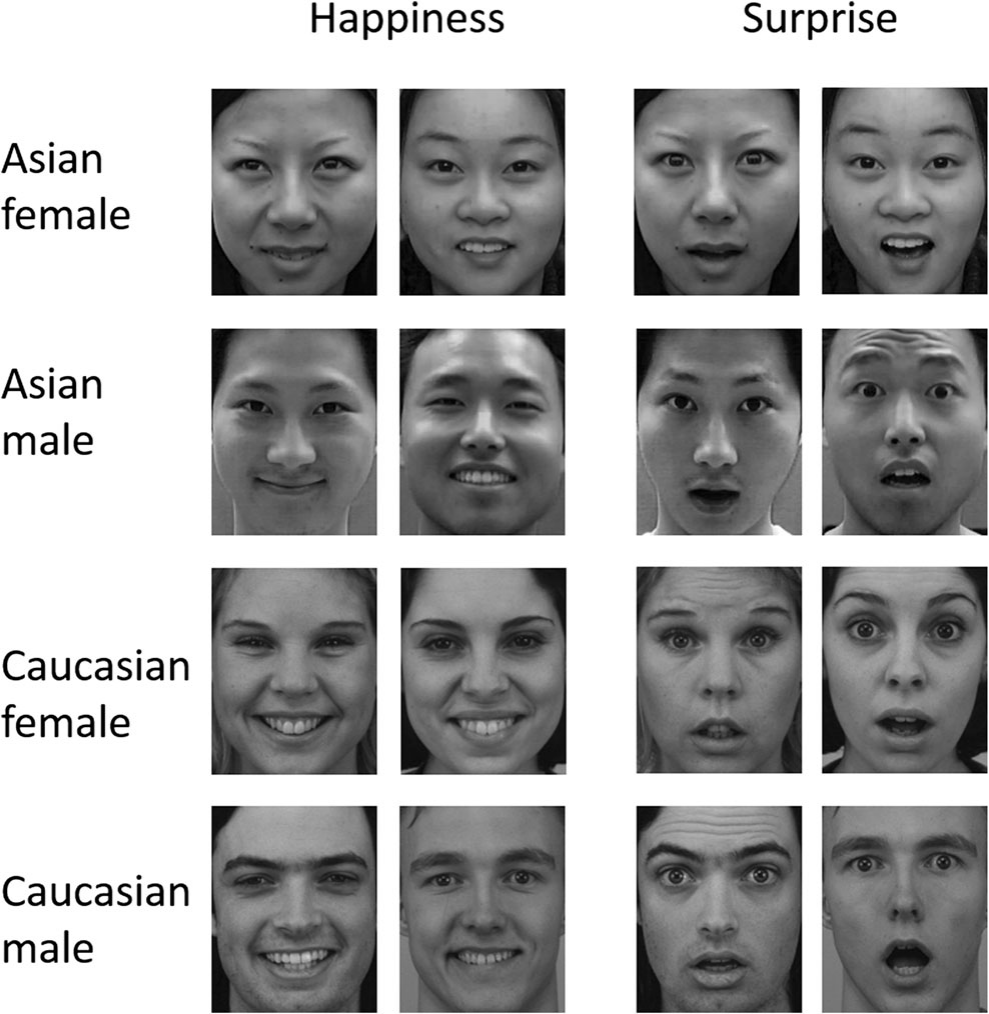
Examples of stimuli used in Experiment 1, with two Asian and two Caucasian female and male models showing happiness and surprise expressions

All face stimuli were cropped around the outline of the face and resized to 300 × 240 pixels and converted to grayscale. When viewed from 57 cm away, each image extended to a visual angle of approximately 7.9 degrees high and 6.4 degrees wide. Figure 1 shows examples of stimuli used in Experiment 1.

### Procedure

**Fig. 2:**
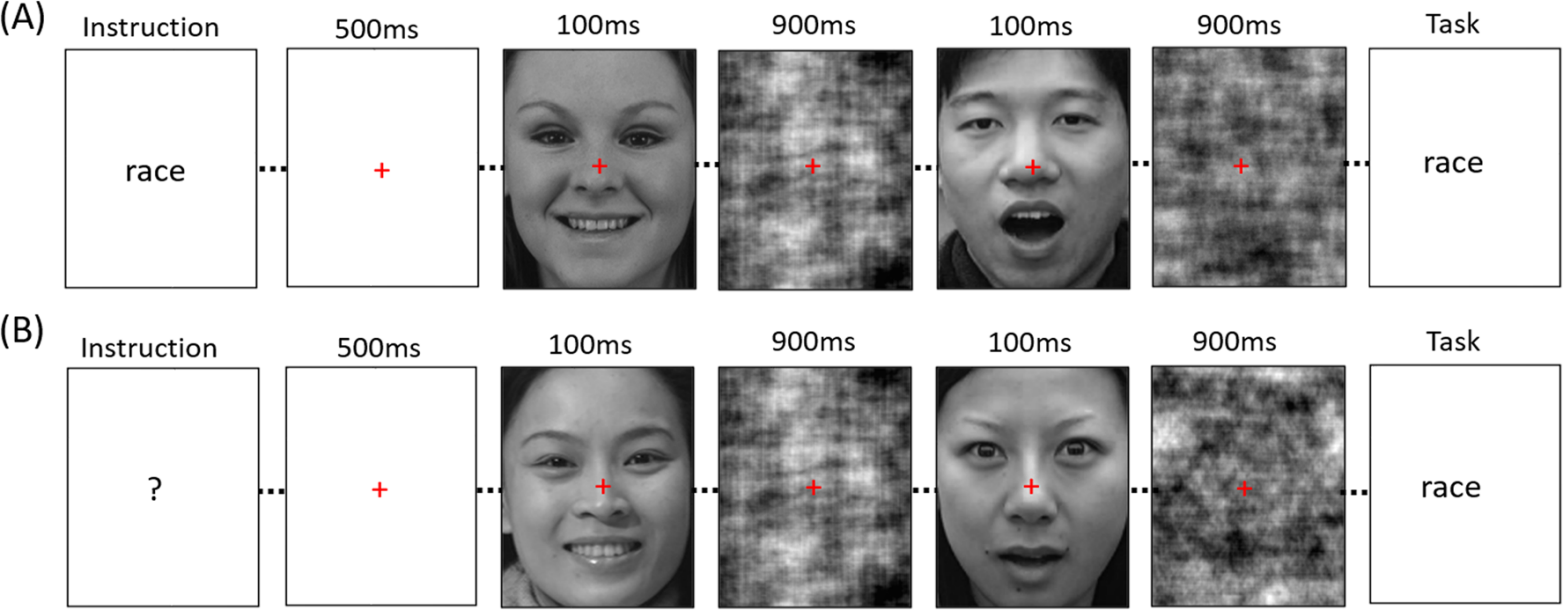
Example trials for before task (**a**) and after task (**b**) instructions. The face pair in the before task instruction example shows a different race–different gender–different identity–different emotion pairing, and the face pair in the after task instruction example shows a same race–same gender–different identity–different emotion pairing. In both of these two example trials, the participant is asked to make a judgment on race

Participants viewed faces presented on a computer screen using PsychoPy (www. psychopy.org). Participants performed a same/different matching task (see Fig. 2). This involved judging whether the two face images were the same or different on one of four characteristics (gender, identity, race, emotion). A central fixation cross was presented throughout each trial. At the beginning of each trial, the fixation cross was presented on a gray background for 500 ms, prior to the presentation of a face image for 100 ms. This was followed by a phase-scrambled mask image for 900 ms and then another face image for 100 ms. Participants were instructed to respond as quickly and as accurately as possible after the second image was presented. On half of the trials, the task instruction (i.e., the characteristic to be judged as same or different) was given at the beginning of the trial, and on half the trials the task instruction was given at the end of the trial. Trials with different tasks (identity, race, gender, emotion) and different instruction timings (before, after) were randomly interleaved.

For judgments of race, gender and emotion, it was possible to vary all nonrelevant dimensions independently. For example, on a race trial, there could be changes in the gender, emotion, or identity of the face that were not relevant to the judgment of race. In contrast, judgments of identity are confounded by changes in gender and identity. For example, if two images differ in gender or race, it is possible to infer that the identity is also different. Thus, for judgments of identity, the only nonrelevant dimension that could be varied was facial expression. We created 20 same trials and 20 different trials for judging each characteristic with the before and after task instruction timings, respectively. Within these trials the nonjudged characteristics were systematically varied to create all possible combinations. Hence (for example), a pair of face images to be judged as having the same expression might vary on gender, identity, or race, and a pair with different expressions might have the same gender, identity, or race. Ten additional trials were also included prior to the main experiment, to form a practice run. The whole experiment took approximately 30 min. In line with standard practice for bounded data, percentage correct values were arcsine transformed before any statistical analysis was performed.

### Results

**Fig. 3:**
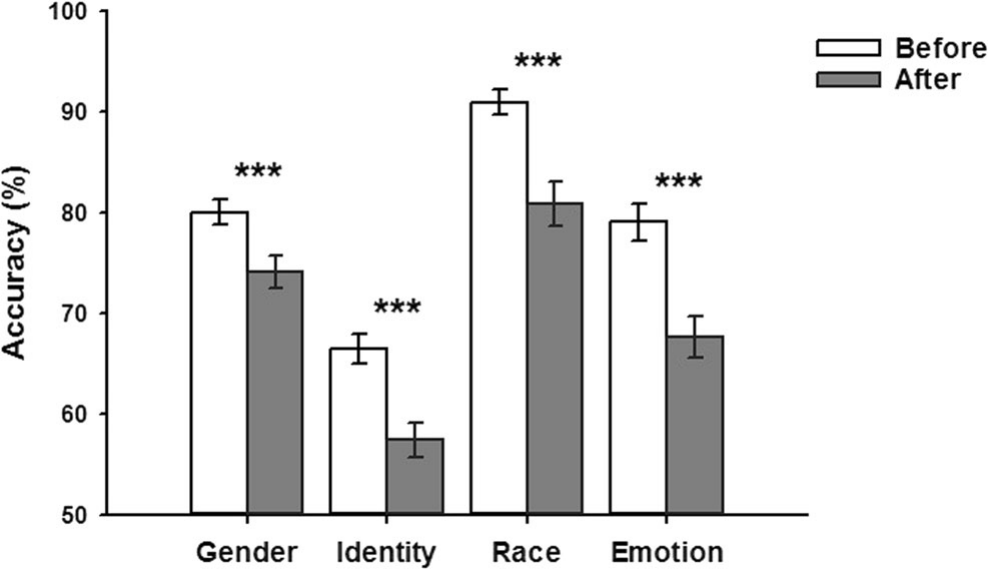
Overall matching accuracies (with error bars) in different tasks for unfamiliar faces in Experiment 1. *Asterisks* denote higher recognition accuracies when the task instruction was given before (rather than after) the face images in the trial. ***p < .001. Chance level performance = 50% correct

Our principal interest is in the accuracy data. The mean accuracy for the different task variants is shown in Fig. 3. A repeated-measures ANOVA was performed on the data with instruction timing (before, after) and task (gender, identity, race, emotion) as within-subjects factors. We found significant main effects of both instruction, *F*(1, 27) = 106.8, *p* < .001, partial η^2^ = 0.80, and task, *F*(3, 81) = 77.4, *p* < .001, partial η^2^ = 0.74. The effect of instruction reflects higher accuracy when the task was given at the beginning of the trial compared to when it was given at the end of the trial. There was also a significant interaction of Instruction × Task, *F*(3, 81) = 4.0, *p* = .01, partial η^2^ = 0.13. This interaction reflects the fact that the effect of instruction was greater for the race task, followed by emotion, identity, and then the gender task. However, for each task, performance was always greater when the instruction was given before the trial: gender, *F*(1, 27) = 14.52, *p* < .001; identity, *F*(1, 27) = 25.80, *p* < .001; race, *F*(1, 27) = 60.47, *p* < .001; emotion, *F*(1, 27) = 32.11, *p* < .001. Finally, we determined whether accuracy was above chance level (0.5) for each condition using a one-sample *t* test. All conditions were significantly above chance: gender-before, *t*(27) = 20.31, *p* < .001; gender-after, *t*(27) = 13.64, *p* < .001; identity-before, *t*(27) = 11.17, *p* < .001; identity-after, *t*(27) = 5.68, *p* < .001; race-before, *t*(27) = 22.61, *p* < .001, race-after, *t*(27) = 12.73, *p* < .001; emotion-before, *t*(27) = 14.07, *p* < .001; emotion-after, *t*(27) = 8.74, *p* < .001.

### Discussion

In this experiment, we investigated the automaticity of unfamiliar face processing across gender, identity, race, and emotional expression. Our strong criterion for automatic processing is that performance is not affected by whether the instruction is given at the beginning or at the end of trial. However, we always found a significant effect of instruction; when the instruction was given at the beginning of a trial, performance was significantly higher than when the instruction was given at the end of the trial. There was nonetheless an interaction between instruction and task, reflecting the fact that the effect of instruction varied across the four tasks. Nevertheless, for all tasks there was a significant effect of instruction, ruling out evidence of automaticity on the strong criterion.

Next, we asked whether the data support the weaker criterion of automaticity, in which accuracy on trials in which the task instruction was given at the end were above chance. Using this weaker criterion, we found evidence of automaticity in the form of above-chance performance for all tasks. Taken together, these results therefore show partial support for the automatic processing of unfamiliar faces, though we note that accuracy in the identity matching condition in particular was not far from chance level when the task instruction was only given at the end of the trial. In fact, the generally poor performance of identity matching with these unfamiliar faces is consistent with other reports of limited ability to judge unfamiliar face identity (e.g., Hancock et al., 2000; Jenkins & Burton, 2011). In Experiment 2 we therefore contrasted automaticity across familiar and unfamiliar faces.

## Experiment 2

The aim of this experiment was to determine the effect of familiarity on automaticity processing of different facial characteristics. This experiment used an equivalent matching task to Experiment 1, involving judgments of gender, identity, race, and emotion. However, we compared performance for familiar and unfamiliar face images. Again, we used two criteria for demonstrating automaticity. Our strong criterion would involve no difference in performance between trials in which the task instruction is given at the beginning or at the end of a trial. Our weaker criterion for automaticity is that performance on a task when the task instruction is given at the end of the trial should be above chance.

### Participants

Twenty-one Caucasian participants from the University of the York were recruited (18 females; *M*_age_, 21.45 years). One participant was removed from analyses because less than 80% (more than eight identities out of 40) of famous face images were recognized. All participants gave their written consent prior to the experiment. The University of York Department of Psychology Ethics Committee approved the study.

### Images

Images of familiar and unfamiliar White and Black faces were obtained from the Internet. There were 10 identities for each combination of race (White or Black), gender (female or male), and familiarity (familiar or unfamiliar) dimensions. In addition, happy and neutral (instead of surprise, for ease of image selection online) face images were selected for each identity. This gave a total of 80 familiar faces and 80 unfamiliar faces. All face stimuli were cropped and resized to 300 × 240 pixels and converted to greyscale. When viewed from 57 cm away, each image extended to a visual angle of approximately 7.9 degrees high and 6.4 degrees wide.

### Procedure

The sequential matching task from Experiment 1 was used, with the following differences: (1) Each face image was presented for 150 ms, and the mask was presented for 850 ms; (2) there were 10 same trials and 10 different trials for each dimension for each before/after task instruction; (3) familiar and unfamiliar trials were randomly interleaved within each block.

We tested the familiarity of the familiar faces for each participant after the matching task was completed. Each participant was presented with a set of images of the familiar faces used in the experiment. None of these images were similar to those used in the main experiment. Participants were asked to write down the name or any relevant identifying information for each face. In this way, we established that participants were able to recognize over 90% of the images of the celebrities used in the experiment. In the main experiment, trials that included a familiar face that was not recognized by a participant during this posttask screening test were not included in the analysis of that participant. Approximately 9% of trials were excluded on this basis.

### Results

**Fig. 4:**
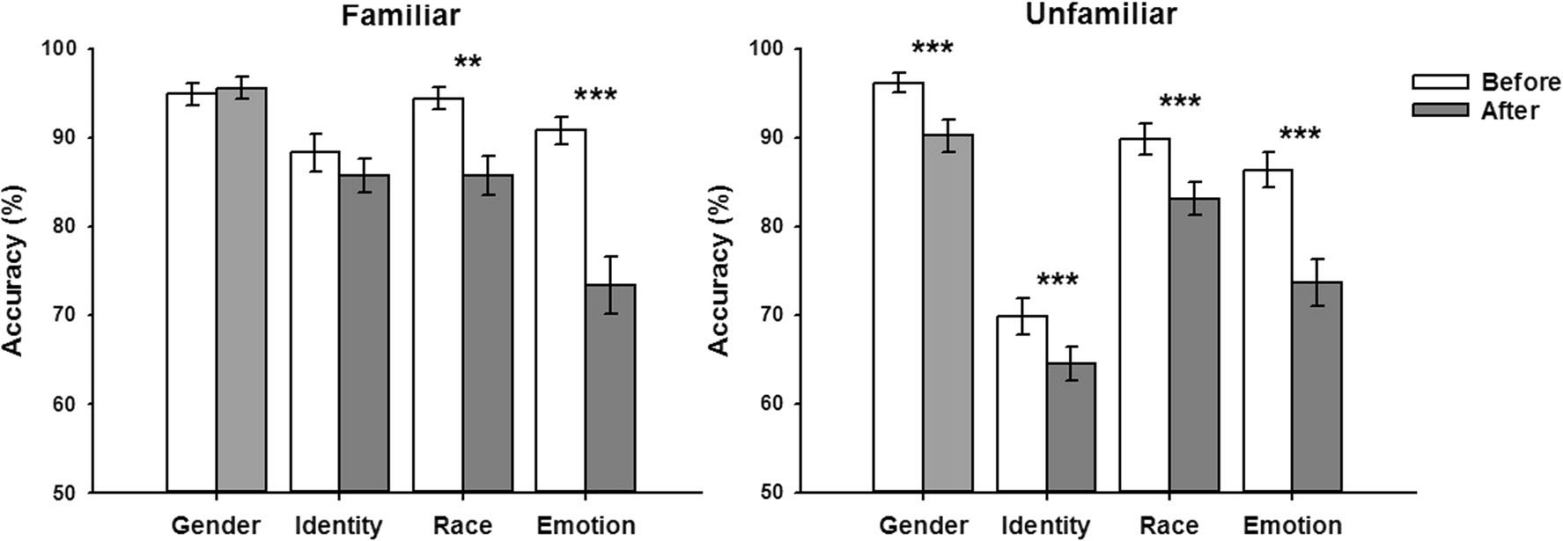
Overall matching accuracies (with error bars) for familiar faces and unfamiliar faces in different tasks in Experiment 2. *Asterisks* denote higher recognition accuracies when the task instruction was given before (rather than after) the face images in the trial. **p < .01, ***p < .001. Chance level performance = 50% correct

The mean accuracy of matching performance for familiar and unfamiliar faces is shown in Fig. 4. A three-way repeated-measures ANOVA was performed, with instruction timing (before, after), face familiarity (familiar, unfamiliar), and task (gender, identity, race, emotion) as within-subjects variables. There were significant main effects of instruction, *F*(1, 19) = 46.54, *p* < .001, partial η^2^ = 0.71; familiarity, *F*(1, 19) = 32.98, *p* < .001, partial η^2^ = 0.63; and task, *F*(3, 57) = 41.0, *p* < .001, partial η^2^ = 0.68. However, there was also a significant three-way interaction of Instruction × Familiarity × Task, *F*(3, 57) = 3.83, *p* < .05, partial η^2^ = 0.17.

To decompose the three-way interaction, separate two-way ANOVAs were conducted for familiar and for unfamiliar faces, with instruction timing (before, after) and task (gender, identity, race, emotion) as within-subjects factors. For familiar faces, there was a main effect of instruction timing, *F*(1, 19) = 16.50, *p* < .001, partial η^2^ = 0.47, and task, *F*(3, 57) = 14.11, *p* < .001, partial η^2^ = 0.43. There was also a significant interaction of Instruction × Task, *F*(3, 57) = 9.23, *p* < .001, partial η^2^ = 0.33. The interaction reflected the fact that there was no effect of instruction timing on some tasks but on other tasks there was better performance when the task instruction given at the beginning of the trial. For example, there was no difference in accuracy for both gender, *t*(19) = 0.46, *p* > .1, and identity, *t*(19) = 1.14, *p* > .1, when the instruction was given before or after the trial. In contrast, accuracy was higher when the instruction was given before the trial in judgments of race, *t*(19) = 3.6, *p* < .01, and expression, *t*(19) = 6.81, *p* < .001. Nonetheless, one-sample *t* tests showed that performance was above chance level (0.5) for all tasks when the instruction was given at the end of the trial: gender-before, *t*(19) = 16.55, *p* < .001; gender-after, *t*(19) = 17.45, *p* < .001; identity-before, *t*(19) = 11.25, *p* < .001; identity-after, *t*(19) = 10.73, *p* < .001; race-before, *t*(19) = 16.32, *p* < .001; race-after, *t*(19) = 12.31, *p* < .001; emotion-before, *t*(19) = 13.82, *p* < .001; emotion-after, *t*(19) = 6.23, *p* < .001.

For unfamiliar faces, there were main effects of instruction timing, *F*(1, 19) = 30.97, *p* < .001, partial η^2^ = 0.62, and task, *F*(1, 19) = 53.3, *p* < .001, partial η^2^ = 0.74. However, there was no significant interaction between Instruction × Task, *F*(3, 57) = 1.65, *p* > .1, partial η^2^ = 0.08. Finally, one-sample *t* tests showed that performance was above chance level (0.5) for all tasks when the instruction was given at the end of the trial: gender-before, *t*(19) = 18.58, *p* < .001; gender-after, *t*(19) = 12.99, *p* < .001; identity-before, *t*(19) = 9.48, *p* < .001; identity-after, *t*(19) = 7.7, *p* < .001; race-before, *t*(19) = 13.40, *p* < .001; race-after, *t*(19) = 12.17, *p* < .001; emotion-before, *t*(19) = 11.91, *p* < .001; emotion-after, *t*(19) = 8.07, *p* < .001.

### Discussion

This experiment investigated automaticity for processing different characteristics of familiar and unfamiliar faces. Consistent with Experiment 1, we found that performance with unfamiliar faces was better when the instruction was given at the beginning of the trial compared to when it was given at the end of the trial. Although unfamiliar faces therefore again failed to meet our strong criterion of automaticity, performance on trials in which the task was given at the end of the trial were above chance, showing that they could satisfy the weak criterion for automaticity. The pattern of results for unfamiliar faces therefore replicates the main findings from Experiment 1.

In contrast, we found an interaction between the effect of instruction timing and task with familiar faces. This reflected the lack of any difference between whether the instruction was given before or after the trial for judgments of gender and identity. This satisfies our strong criterion for automaticity of gender and identity perception with familiar faces. However, we found that performance on judgments of the race or expression of familiar faces was higher when the task was given before the trial; for these characteristics, only the weaker criterion of above-chance performance when the instruction was given at the end of the trial was met. These results show that the familiarity of the faces does influence the automaticity with which they are processed but that this influence is more pronounced for judgments of gender and identity.

### General discussion

The aim of this study was to investigate whether face perception is automatic and to what extent this varies across different facial characteristics. Participants performed a matching task on two sequentially presented faces. Across trials, faces varied randomly in four dimensions: race, gender, identity, and emotion. At the end of each trial, participants had to make a same/different judgment on one of the dimensions.

As a strong test for automaticity, we compared the responses to trials when the instruction for the trial was given at the beginning with trials in which the instruction was given at the end. If perception of a particular characteristic is fully automatic, we reasoned that there should be no difference in accuracy whether the task was given at the beginning or the end of the trial. In Experiment 1, however, we found higher accuracy with unfamiliar faces across all dimensions when the instruction was given at the beginning of the trial, showing a lack of full automaticity. Unfamiliar faces only met weaker criterion for automaticity of above-chance performance when the instruction was given at the end of the trial.

In Experiment 2, we asked how familiarity influences automatic processing. Consistent with Experiment 1, accuracy with unfamiliar faces was higher across all dimensions when the instruction was given at the beginning of the trial; these findings with unfamiliar faces again did not meet our strong criterion for automatic processing. However, for familiar faces, the accuracy of gender and identity judgments was not affected by the timing of the task instruction. Nonetheless, race and expression judgments with familiar faces still only met our weaker criterion for automaticity of above-chance performance in the delayed instruction condition. Taken together, these findings show that while the processing of unfamiliar faces is not fully automatic, familiarity with a face increases automaticity for certain facial characteristics.

Our findings are relevant to current theories on the social categorization of faces. Categories such as race and gender can largely be determined from purely visual facial properties (Kramer, Young, Day, & Burton, 2017), and some theories therefore suggest that their extraction may involve entirely bottom-up, preattentive, and automatic processes (Bargh, 1997; Brewer, 1988; Fiske & Neuberg, 1990; Freeman & Ambady, 2011). However, other theories maintain that there is also some top-down control of social categorization (Macrae & Bodenhausen, 2000). Our findings suggest that an interaction between bottom-up and top-down processes can explain the categorization of unfamiliar faces. We found that performance on all facial judgments with unfamiliar faces was above chance even when the characteristic that had to be matched was not given until the end of the trial. This demonstrates a clear contribution from bottom-up processing of different facial dimensions. Moreover, this bottom-up processing showed some limited form of automaticity because participants did not know to which aspect of the images they should attend until after both images were presented. However, we also found that performance with unfamiliar faces was always better when the task was given at the beginning of the trial, which provides a clear demonstration of the involvement of top-down control.

The majority of previous studies on automaticity have used unfamiliar faces. However, it is well-established that familiar faces are processed more effectively than unfamiliar faces (Burton et al., 2005; Hancock et al., 2000; Jenkins & Burton, 2011). In Experiment 2, we compared automatic processing of familiar and unfamiliar faces. Critically, we found an interaction between the timing of instruction, facial dimension and familiarity. This interaction was explained by the fact that judgments of gender and identity from familiar faces were not different when the instruction was given at the beginning compared to the end of the trial. These findings meet our strong criterion for automaticity and are consistent with previous work that has shown fewer attentional resources are required to detect familiar compared to unfamiliar faces (Gobbini et al., 2013; Jackson & Raymond, 2006; Tong & Nakayama, 1999; Visconti di Oleggio Castello & Gobbini, 2015).

Interestingly, familiarity did not lead to strongly automatic processing of all facial dimensions. Accuracy on race and emotion trials was significantly lower when the task instruction was given at the end of the trial. It makes sense that familiarity helps with judgments of facial identity, as it is known that judgments of identity are much easier to make with familiar compared to unfamiliar faces (Hancock et al., 2000; Jenkins et al., 2011; Jenkins & Burton, 2011; Davies-Thompson et al., 2013; Young & Burton, in press). Familiarity with a face also allows us to retrieve a range of semantic information associated with a person. The effect of familiarity on the automaticity of gender judgments may therefore suggest either that the perceptual representation of identity is linked to the perceptual representation of gender or that the automatic recognition of familiar face identity allowed participants to infer the gender. In this respect, it was surprising that familiarity did not influence the automaticity of race judgments. Although it may seem intuitive that a person’s identity should be tightly linked to our representation of their race, previous studies have suggested that this is not always the case. For example, Phelps and colleagues (2000) showed that the difference in the amygdala response to Black compared to White unfamiliar faces could be predicted by implicit measures of racial attitude, yet a similar effect was not evident when familiar Black and White faces are evaluated. Likewise, behavioral studies show that familiar other-race faces do not show characteristics that are often associated with the classic other-race effect (McKone, Brewer, MacPherson, Rhodes, & Hayward, 2007). This suggests that when a face becomes familiar the neural representation of identity may become dissociated from the representation of race. We note, though, that despite the steps taken to establish that participants could recognize the familiar faces with unlimited presentation time, some errors were nonetheless made in the identity matching task, making it clear that the familiar faces were not always successfully recognized with the brief masked presentations. It is therefore also possible that these failures in recognition might have influenced performance in the race classification task.

From some perspectives, it is perhaps not surprising that face familiarity did not confer any advantage in judgments of facial expression. Since the idea was put forward by Bruce and Young (1986), models of face processing have suggested that invariant properties of the face, such as identity, are processed independently of changeable properties such as expression (Andrews & Ewbank, 2004; Duchaine & Yovel, 2015; Haxby et al., 2000; Young & Bruce, 2011). Against this mainstream opinion, however, there have been reviews that have pointed to limitations in the available evidence (Calder & Young, 2005) and findings that point to some interaction between familiar face identity and expression (Kaufmann & Schweinberger, 2004; Martens, Leuthold, & Schweinberger, 2010). Our findings of clear differences between the automaticity of judgments of identity and expression for familiar faces therefore have important implications as they imply substantial separation between the processing of identity and expression.

In conclusion, this study has developed a novel behavioral paradigm to investigate the automaticity of face perception. We found evidence for partial automaticity in the processing of unfamiliar faces. However, for all dimensions tested (gender, identity, race, emotion), there was evidence of significant top-down control. In contrast to unfamiliar faces, we found full automaticity for judgments of gender and identity to familiar face, whereas judgments of race and emotion were again only partially automatic. These results demonstrate the importance of familiarity in the automaticity of face perception but show that familiarity has differential effects on perceiving different facial characteristics.
